# Uremia causes premature ageing of the T cell compartment in end-stage renal disease patients

**DOI:** 10.1186/1742-4933-9-19

**Published:** 2012-09-12

**Authors:** Ruud WJ Meijers, Nicolle HR Litjens, Elly A de Wit, Anton W Langerak, Ashley van der Spek, Carla C Baan, Willem Weimar, Michiel GH Betjes

**Affiliations:** 1 Department of Internal Medicine, section Nephrology and Transplantation, Erasmus Medical Center, Rotterdam, the Netherlands; 2 Transplantation, Erasmus Medical Center, Rotterdam, the Netherlands; 3 Department of Immunology, Erasmus Medical Center, Rotterdam, the Netherlands

**Keywords:** Ageing, CD28null, End-stage renal disease, Renal replacement therapy, T lymphocytes, Uremia

## Abstract

**Background:**

End-stage renal disease (ESRD) patients treated with renal replacement therapy (RRT) have premature immunologically aged T cells which may underlie uremia-associated immune dysfunction. The aim of this study was to investigate whether uremia was able to induce premature ageing of the T cell compartment. For this purpose, we examined the degree of premature immunological T cell ageing by examining the T cell differentiation status, thymic output via T cell receptor excision circle (TREC) content and proliferative history via relative telomere length in ESRD patients not on RRT.

**Results:**

Compared to healthy controls, these patients already had a lower TREC content and an increased T cell differentiation accompanied by shorter telomeres. RRT was able to enhance CD8^+^ T cell differentiation and to reduce CD8^+^ T cell telomere length in young dialysis patients. An increased differentiation status of memory CD4^+^ T cells was also noted in young dialysis patients.

**Conclusion:**

Based on these results we can conclude that uremia already causes premature immunological ageing of the T cell system and RRT further increases immunological ageing of the CD8^+^ T cell compartment in particular in young ESRD patients.

## Background

Loss of renal function is related to impaired function of the T cell-mediated immune system. Changes in T cell subsets and function may underlie this effect [1,2]. Clinical consequences of this T cell-mediated immune dysfunction are a reduced efficiency of vaccination [3,4], an enhanced susceptibility for infectious diseases [5] and an enchanced risk for developing auto-immune diseases and tumors [6].

T cells leave the thymus as naïve cells. Upon encountering of antigens presented by antigen presenting cells, naive T cells will differentiate into effector T cells and eventually only a fraction of these will develop into memory T cells. The expression of (chemokine C-C motif receptor 7) CCR7 and CD45RO can be used to distinguish between the different T cell subsets, i.e. naïve (CD45RO^-^CCR7^+^), central memory (CM, CD45RO^+^ CCR7^+^, able to home into lymphoid tissues), effector memory (EM, CD45RO^+^CCR7^-^, exerting direct effector functions) and the more terminally differentiated effector memory CD45RA^+^ (EMRA, CD45RO^-^CCR7^-^, high in effector function) subset [2,7,8]. In addition, the loss of cell surface CD28 expression identifies more differentiated T cells [9].

During ageing in healthy individuals, the thymic output of new naïve T cells reduces due to the involution of the thymus. Absolute T cell numbers are largely conserved by homeostatic proliferation of both naïve and memory T cells but eventually this leads to a reduced population of naïve T cells and a relatively preserved population of memory T cells [10]. Elderly individuals have a marked decrease in naïve T cells,, a decline in CD4/CD8 ratio and a relative increase in the number of differentiated memory T cells lacking CD28 [10-12].

The thymic output of new naïve T cells can be determined by measuring the T cell receptor excision circles (TRECs)[13]. These TRECs are small circular DNA episomes that are formed during rearrangement of the T cell receptor (TCR) genes in T cells that are present in the thymus. These TRECs are not replicated and therefore diluted with every cell division. Another hallmark of ageing is the reduction in telomere length [14,15]. Telomeres are small DNA sequences located at the end of a chromosome and with increasing age they become shorter due to the inability of telomerase to elongate these DNA sequences upon division. Together, TREC content and telomere length reflect the thymic output and replicative history of T cells and may provide a valuable tool to estimate the immunological age of the T cells within an individual.

Using these ageing parameters, the T cell system of a dialysis patient shows severe T cell ageing and resembles that of a 20–30 year older healthy individual [1]. This premature ageing of the T cell system probably underlies the uremia-associated immune defect in dialysis patients. However, it is not known whether the T cell system of ESRD patients not on renal replacement therapy (RRT) shows a similar degree of immunological ageing and to what extent this is influenced by RRT.

In this cross-sectional study, we have analyzed the T cell ageing parameters in ESRD patients who are not on RRT and compared this to healthy individuals on one hand, and patients treated with RRT (i.e. hemodialysis or peritoneal dialysis) on the other hand.

## Results

### T cell ageing parameters in hemodialysis and peritoneal dialysis patients

Initially, T cell numbers and ageing parameters of ESRD patients treated with hemodialysis were compared to peritoneal dialysis patients but no statistically significant differences were observed. Therefore, the data of these two RRT groups were combined for comparison with the data of ESRD patients not on RRT (non-RRT group). The clinical and demographic characteristics of patients and healthy controls are shown in Table 1. Compared to the old ESRD patient group, the young patients showed a different distribution of underlying kidney diseases (less frequently hypertensive nephropathy and more frequently reflux nephropathy). Moreover, the young RRT group had a longer history of dialysis treatment than the old RRT group. However, type of underlying kidney disease and dialysis vintage were not significantly associated with any of the ageing parameters measured.

**Table 1:** Study population characteristics

	**End-stage renal disease patients not receiving renal replacement therapy**		**End-stage renal disease patients receiving renal replacement therapy**		**Healthy controls**	
Group	Young	Old	Young	Old	Young	Old
Number of individuals	22	33	49	49	55	65
Age in Years	33.5 ± 9.6^*^	64.4 ± 6.3^*^	35.7 ± 8.5^*^	63.1 ± 8.7^*^	38.4 ± 7.9^*^	63.5 ± 7.5^*^
Male	77.3%	60.6%	55.1%	71.4%	49.1%	38.5%
CMV positive	54.5%	51.2%	59.2%	53.1%	50.9%	49.2%
Hemodialysis			25	32		
Peritoneal dialysis			24	17		
Duration of RRT (years)			4.30 (0.1 - 22)^**^	2.76 (0.2 - 18)^**^		
*Underlying kidney disease*						
- Hypertensive nephropathy	13.6%	27.2%	20.8%	30.6%		
- Primary glomerulopathy	31.8%	18.2%	18.8%	18.4%		
- Diabetic nephropathy	0%	21.2%	2.1%	8.2%		
- Polycystic kidney disease	9.1%	9.0%	0%	2.0%		
- Reflux nephropathy	18.2%	3.0%	14.6%	4.1%		
- Other	9.1%	15.2%	20.9%	8.2%		
- Unknown	18.2%	6.0%	22.9%	28.6%		

* Data are given in means with standard deviation.

** Data are given in means with range.

### CD4^+^ T cell differentiation is increased in ESRD patients but marginally affected by RRT

**Figure 1:**
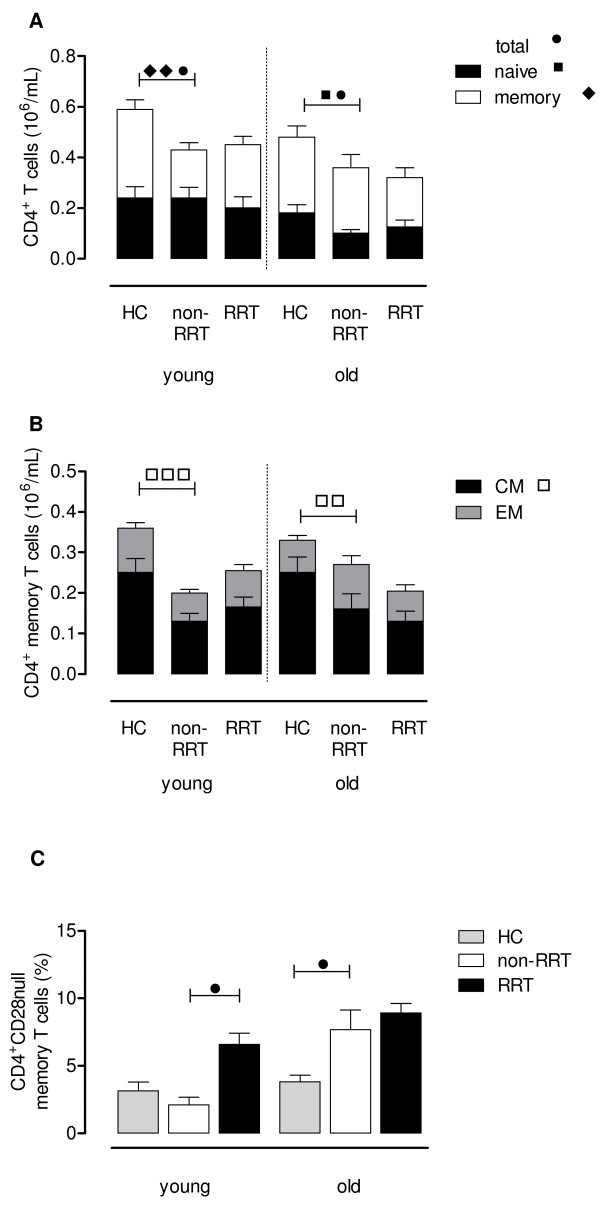
**CD4**^**+**^**T cell phenotype and differentiation status.** Using a whole blood staining, the phenotype and differentiation status of CD4^+^ T cells were determined in HC and ESRD patients not on RRT or receiving RRT. The absolute number of CD4^+^ T cells of young (age: <50 years) and old (age: ≥50 years) HC and these ESRD patients was dissected into a naïve (closed bars) and memory (open bars) compartment (**A**). Next, the composition of the memory compartment (CM in black and EM in grey bars) of CD4^+^ T cells is given for young and old HC and ESRD patients on RRT or not (**B**). In addition, we also determined the percentage memory CD4^+^ T cells lacking CD28 (i.e. CD28null) on their cell surface as another marker for T cell differentiation for HC (grey bars) and ESRD patients not on RRT (white bars) or receiving RRT (black bars) (**C**). Bars represent the means + SEM and statistically significant differences between the groups are shown (one symbol: p < 0.05, two symbols: p < 0.01, three symbols: p < 0.001).

The young and old non-RRT groups had significant lower numbers of CD4^+^ T cells when compared to age-matched Healthy Controls (HC). In the young non-RRT group, a lower absolute number of memory T cells and comparable numbers of naïve T cells were observed when compared to that of age-matched HC. Especially in the old non-RRT group, the CD4^+^ T cells were shifted towards the memory phenotype with significant less naïve T cells when compared to age-matched HC (Figure 1A). The CD4^+^ memory compartment of the non-RRT group contained significantly (p < 0.01) lower numbers of central memory T cells, resulting in a relative increase of more differentiated effector-memory T cells when compared tot that of age-matched HC (Figure 1B). No differences were observed if RRT patients were compared to age-matched non-RRT patients, with respect to the composition of the total CD4^+^ T cell population (Figure 1A) and differentiation of memory CD4^+^ T cells (Figure 1B).

Elderly, but not young, non-RRT patients had a more differentiated memory phenotype when compared to that of age-matched HC (Figure 1C) based on the percentage of CD28null memory T cells (7.69 ± 1.46% versus 3.83 ± 0.49%, p < 0.05). RRT only resulted in a significant (p < 0.05) higher percentage of CD4^+^CD28null memory T cells in the young patient group (Figure 1C).

### CD8^+^ T cell differentiation is increased and significantly different in the young RRT patients

**Figure 2:**
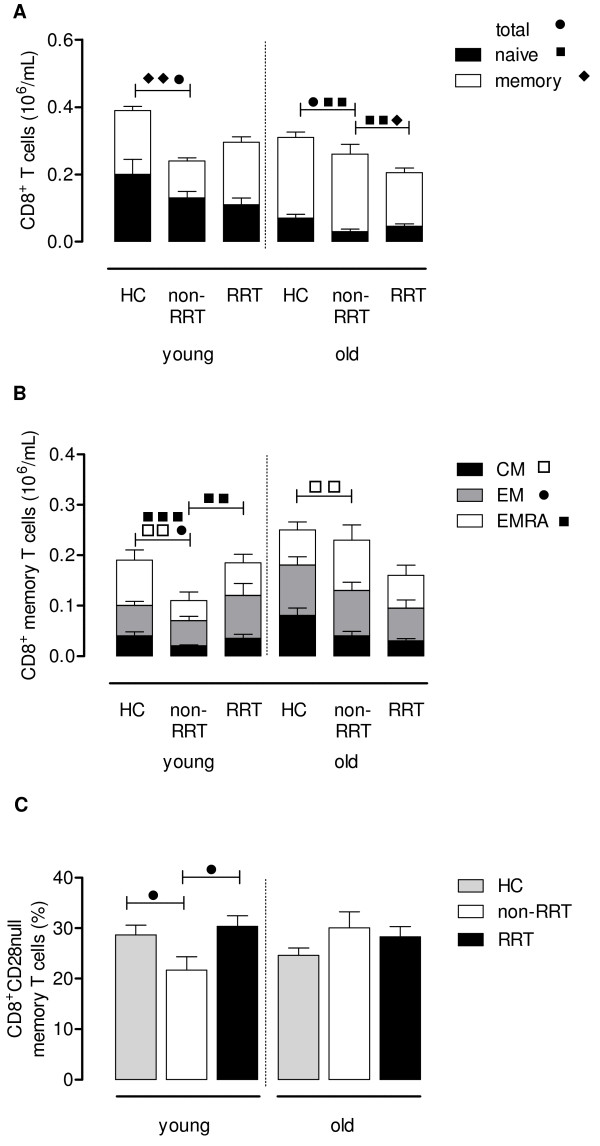
**CD8**^**+**^**T cell phenotype and differentiation status.** Using a whole blood staining, the phenotype and differentiation status of CD8^+^ T cells were determined in HC and ESRD patients not on RRT or receiving RRT. The absolute number of CD8^+^ T cells of young (age: <50 years) and old (age: ≥50 years) HC and these ESRD patients was dissected into a naïve (closed bars) and memory (open bars) compartment (**A**). Next, the composition of the memory compartment (CM in black, EM in grey and EMRA in white bars) of CD8^+^ T cells is given for young and old HC and ESRD patients on RRT or not (**B**). In addition, we also determined the percentage memory CD8^+^ T cells lacking CD28 (i.e. CD28null) on their cell surface as another marker for T cell differentiation for HC (grey bars) and ESRD patients not on RRT (white bars) or receiving RRT (black bars) (**C**). Bars represent the means + SEM and statistically significant differences between the groups are shown (one symbol: p < 0.05, two symbols: p < 0.01, three symbols: p < 0.001).

Compared to the CD4^+^ T cell compartment, the number and differentiation of circulating CD8^+^ T cells was more affected in non-RRT patients. On average, absolute numbers of CD8^+^ T cells were lower compared to age-matched HC which was largely attributable to a significant decrease in memory T cells in young non-RRT patients, and decreased naïve T cell numbers in the old non-RRT group (Figure 2A). Comparing old non-RRT patients to age-matched RRT patients revealed a slight but significantly (p < 0.05) decreased number of naïve and memory T cells (Figure 2A).

The CD8^+^ memory T cell compartment of the young and old non-RRT groups consisted of significantly (p < 0.01) less CM when compared to age-matched HC, resulting in relatively more differentiated CD8^+^ T cells with a EM/EMRA phenotype (Figure 2B). The influence of RRT was only observed within the young RRT group. These younger patients receiving RRT showed an increased differentiation of the memory CD8^+^ T cell compartment, reflected by an increase in EMRA T cell numbers (p < 0.01, Figure 2B) compared to non-RRT patients. This finding was in accordance with the increased percentage of memory CD8^+^ T cells lacking CD28 on their cell surface as compared to the group of young non-RRT patients (p < 0.05, Figure 2C).

### Thymic output of T cells and relative telomere length of CD4^+^ and CD8^+^ T cells

**Figure 3:**
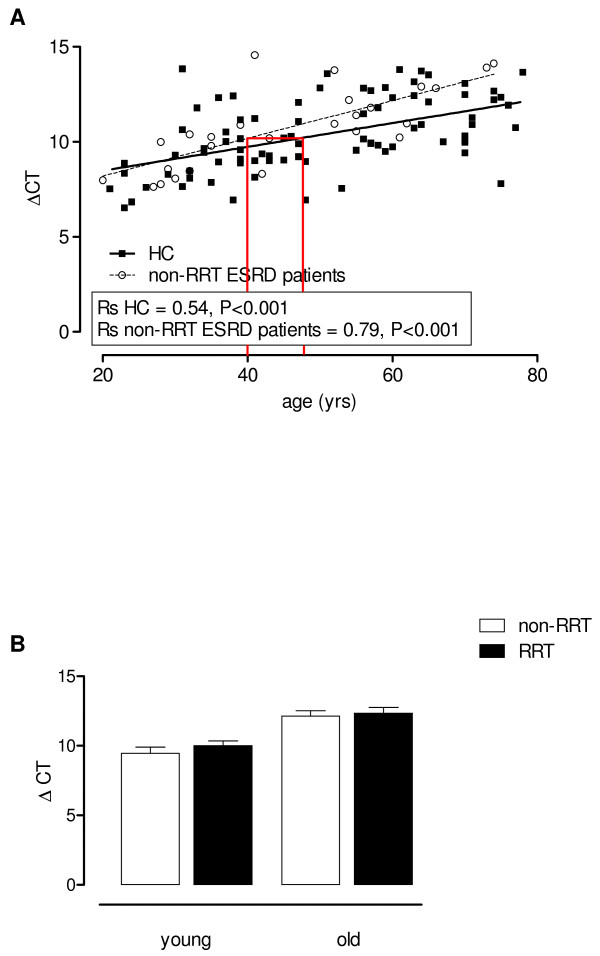
**Quantification of the TREC content by quantitative PCR.** The threshold cycle (Ct) is the number of amplification cycles needed to detect the TRECs and is a relative measure, inversely related with the concentration of TRECs. Control for DNA input was done by performing a quantitative PCR (qPCR) for albumin and the difference between the Ct for TRECs and the Ct for albumin was calculated (ΔCt). The ΔCt, indicative for TREC content, was determined of HC (straight line) and ESRD patients not on RRT (dotted line) and lines were compared using a linear regression analysis (p < 0.05 for the difference between lines for ESRD patients not on RRT and HC). The lines in (**A**) mark the discrepancy between the calendar age (i.e. 40 years) of a non-RRT patient and the immunological age, by extrapolation of the value for the deltaCT (TREC content) to the HC regression line. In addition, Spearmans Rho correlation coefficients (Rs) were calculated for HC and ESRD patients not on RRT to determine the strength of the association between TREC content (calculated as ΔCt) and age. Age is depicted on the X-axis whereas on the Y-axis the ΔCt value for TREC content is displayed. Next, the effect of RRT on the TREC content (**B**) was analyzed by dissecting the ESRD patients into a young (<50 years) and old (≥50 years) group and either not (white bars) or receiving (black bars) RRT. Bars represent means + SEM.

In young and old non-RRT patients a lower thymic T cell output was shown by the significant lower TREC content of T cells compared to age-related HC (Figure 3A, p < 0.05) for comparison of the two lines. Using linear regression analysis for both HC as well as non-RRT patients, an average immunological age for a non-RRT patient with a calendar age of 40 years was estimated. For this purpose, the value for thymic output (deltaCt, TREC content) of this 40 year old patient was calculated using the formula for the regression line of non-RRT patients. This value was then plotted in the formula for the regression line of HC and resulted in an average calendar age of 46.6 years. The immunological age of the patient’s T cells using thymic output was thus increased with approximately 7 years (Figure 3A), lines indicate the discrepancy between calendar ages of a non-RRT patient and a HC (i.e. the immunological age of the non-RRT patient), respectively. However, the TREC content was equally low for non-RRT and RRT patients (Figure 3B).

**Figure 4:**
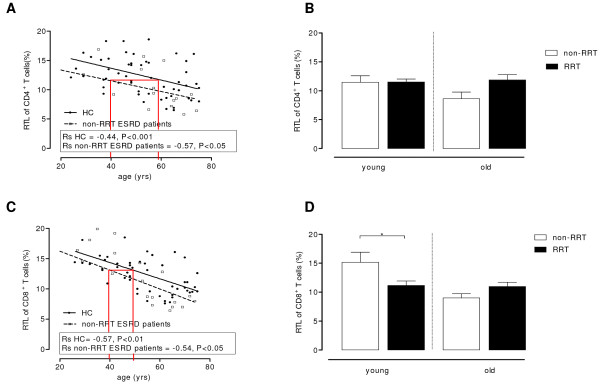
**Relative telomere length of CD4**^**+**^**and CD8**^**+**^**T cells.** The relative telomere length (RTL) of the CD4^+^ (**A**) and CD8^+^ (**C**) T cells is determined of HC (straight line) and ESRD patients not on RRT (dotted line) and lines were compared using a linear regression analysis i.e. p < 0.05 and p = 0.07 when comparing the differences between HC and non-RRT patients with respect to CD4^+^ RTL and CD8^+^ RTL respectively. The lines in A and C highlight the discrepancy between the calendar age (i.e. 40 years) of a non-RRT patient and the immunological age, by extrapolation of the value for the CD4^+^ and CD8^+^ RTL to the HC regression line, respectively. In addition, Spearmans Rho correlation coefficients (Rs) were calculated for HC and ESRD patients not on RRT to determine the strength of the association between RTL and age. Age is depicted on the X-axis and the RTL on the Y-axis. The effect of RRT on the RTL of CD4^+^ (**B**) as well as CD8^+^ (**D**) T cells was analyzed by dissecting the ESRD patients into a young (<50 years) and old (≥50 years) group and either not receiving RRT (white bars) or receiving RRT (black bars). Bars represent means + SEM. Statistical differences between the groups are shown (* p < 0.05).

The relative telomere length (RTL) of CD4^+^ (Figure 4A) as well as CD8^+^ T cells (Figure 4C) decreased in both HC and non-RRT patients with increasing age, although these patients had or tendet to have shorter telomeres within both CD4^+^ T cells (p < 0.05, Figure 4A) and CD8^+^ T cells (P = 0.07, Figure 4C) when compared to age-matched HC. Using regression analysis for these ageing parameters, as described above, the immunological age of a 40-year old non-RRT patient amounted to approximately 60 years. A significant (p < 0.05) lower RTL for CD8^+^ (11.17 ± 0.74% versus 15.15 ± 1.73%, Figure 4D), was found in the young RRT group when compared to the non-RRT group. No differences in RTL were observed for the CD4^+^ T cells between RRT and non-RRT groups (Figure 4B).

## Discussion

The results of this study show that based on several immunological parameters, ESRD without RRT is associated with premature immunological ageing of the T cell system. The added effect of RRT on this phenomenon is remarkably small and was predominantly limited to the CD8^+^ T cell compartment in young ESRD patients.

Most studies on the immune system of patients with renal failure have been performed in chronic ESRD patients treated with RRT, mainly hemodialysis. Lymphopenia and signs of T cell activation have been reported in a lot of the studies [16,17]. In-depth analysis of T cell subsets showed that lymphopenia is particularly prominent in the naïve T cell subset which showed a progressive decline in numbers as the stage of chronic kidney disease increased [2,18]. In a recent study, we showed that decreased numbers of naïve T cells in hemodialysis patients is related to decreased thymic output of naïve T cells with increased but insufficient homeostatic proliferation in the periphery [19]. Memory T cells were in general more differentiated probably due to increased proliferation, given the decrease in relative telomere length. Similar findings were now observed for ESRD patients not on dialysis, indicating that loss of renal function is the most dominant factor for decreased thymic output of naïve T cells and increased differentiation/proliferation of memory T cells. In healthy individuals these changes are observed in the elderly and therefore considered as a physiological process of immunological ageing of the T cell system. In comparison to the CD8^+^ T cells, the CD4^+^ T cell system in healthy individuals remains relatively unaffected by age until the seventh or eighth decade [10,12,20-23]. However, patients with ESRD not on RRT already showed all characteristics of immunological ageing (lower thymic output, shorter telomeres) of their T cell system, approximately 10–20 years ahead of their calendar age. The reasons for premature T cell ageing in patients with chronic renal failure are not known but a relative lack of the T cell growth factor IL-7 has been documented and may be important [1,2]. In animal models it was clear that sudden loss of renal function causes involution of the thymus and other lymphoid organs confirming a direct relationship between kidney function and lymphopoiesis [24]. Lymphopenia may trigger increased homeostatic proliferative responses [25], not only of the circulating naïve T cell compartment but also of memory T cells thereby inducing differentiation and loss of telomere length. However this concept is hypothetical and has not been tested yet in ESRD patients.

In general, immunological ageing of T cells (e.g. increased numbers of CD28null T cells) has been associated with decreased T cell immunity. Maintenance and generation of a number of antibody responses seems critically dependent on the presence of antigen-specific CD4^+^ T cells [3,26]. Any major disturbances of the T cell system will therefore affect the humoral immune response as well. ESRD-related premature immunological T cell ageing may therefore underlie the well-established uremia-associated cellular and humoral immune deficiency in ESRD patients [1].

In a previous study, it was shown that loss of naïve T cells and increased memory T cell differentiation progresses with increasing stage of chronic kidney disease but with little difference between ESRD patients with or without RRT [2,18]. Also on the level of T cell chemokine receptor expression, which is indicative for functional capacities of T cells few differences were observed between T cells of ESRD patient with or without RRT. These findings are remarkable as most patients on dialysis have little to no residual renal function and as such are metabolically more affected than ESRD patient not on dialysis. The results in this study are largely in accordance with these data and show that patients with RRT do not have an altered thymic output of naïve T cells and total numbers of naïve T cells. Summarizing the present data, it appears that the maximum effect of loss of renal function on the T cell immune system is reached at the level of ESRD. Whether RRT prevents further immunological ageing or not is difficult to assess and cannot be inferred from our data.

However, the CD4^+^ and CD8^+^ memory T cells in young dialysis patients showed more differentiation and in the CD8^+^ T cells this was accompanied by a decrease in RTL. Thus, particularly memory CD8^+^ T cells in younger patients show a history of more proliferation without the presence of decreased thymic output. This finding indicates that in younger dialysis patients, on top of ESRD-related immunological ageing, other factors drive the proliferation of memory T cells. The dialysis vintage of younger patients was on average higher than the old group suggesting a role for duration of RRT and loss of telomere length. However, we could not find an independent statistically significant relation between duration of RRT and any of the immunological parameters measured. In addition, the type of underlying kidney diseases was not related to any parameter of immunological ageing. Another possible scenario may be that RRT actually improves homeostatic proliferation of memory T cells in the young but not in the elderly patients. However, this should result in a relative increase in memory T cell numbers in the young RRT patients compared to ESRD patients without RRT which was not observed. The lack of such a finding would argue against this explanation.

A limitation of the present study is the cross-sectional design which may obscure subtle changes in immunological T cell ageing after patients with ESRD have started RRT. However, the large number of patients included in this study adds to the reliability of the results.

In conclusion, severe loss of renal function leading to ESRD is a very potent inducer of premature immunological T cell ageing of both the CD4^+^ and CD8^+^ T cells. Renal replacement therapy is associated with a small increase of memory T cell ageing in patients <50 years of age, particularly in the CD8^+^ T cell subset. Further research is needed to establish the pathophysiology of ESRD-related T cell ageing and whether this can be reversed by e.g. interleukin-7 therapy [27,28] or kidney transplantation.

## Methods

### Study population

ESRD patients were defined by a glomular filtration rate (GFR) of ≤ 15 ml/min and were either not on RRT or treated with hemodialysis or peritoneal dialysis. Patients having an infection, malignancy, autoimmune disease or a history of immunosuppressive drugs (including previous kidney transplantations) were excluded. Healthy individuals were included as controls. They were matched for age and cytomegalovirus (CMV) positivity, as these are well-known factors affecting the composition of the T cell compartment [1,19]. The clinical and demographic characteristics of the ESRD patients and HC are shown in Table 1. All individuals included gave informed consent and the local medical ethical committee approved the study (METC number: 2012–022). It was conducted according to the principles of Declaration of Helsinki and in compliance with International Conference on Harmonization/Good Clinical Practice regulations.

### Differentiation status of circulating T cells

T cell phenotype and differentiation status was analyzed as described previously [1,19]. Briefly, whole blood was stained with AmCyan labeled anti-CD3 (BD Biosciences, Erembodegem, Belgium) in combination with pacific blue labeled anti-CD4 (BD) or allophycocyanin Cy7 (APC-Cy7) labeled anti-CD8 (BD) to identify CD4^+^ or CD8^+^ T cells that are further dissected into four different subsets based on the expression of CCR7 and CD45RO using fluorescein isothiocyanate (FITC) labeled anti-CCR7 (R&D systems, Uithoorn, The Netherlands) and allophycocyanin (APC) labeled anti-CD45RO (BD). Naive T cells are CCR7^+^ and CD45RO^-^, Central memory (CM) cells are CCR7^+^ and CD45RO^+^, Effector memory (EM) cells are CCR7^-^ and CD45RO^+^ and EMRA cells are CCR7^-^ and CD45RO^-^.

T cell differentiation is associated with loss of CD28 expression on cell surface. Percentages of CD28^-^ (or CD28null) T cells within the T cell subsets were determined by staining with peridinin chlorophyll-Cy5.5 (PerCP-Cy5.5) labeled anti-CD28 (BD).

### PBMC isolation

Peripheral blood mononuclear cells (PBMC) were isolated from heparinized blood samples by Ficoll gradient centrifugation. In hemodialyis patients, the blood samples were drawn before a hemodialysis session [1]. Two million PBMC were snap-frozen for the TREC assay and the rest of the PBMC were frozen in liquid nitrogen with a minimum amount of 10 × 10^6^ cells per vial for further experiments.

### DNA isolation and TREC assay

TREC content was assessed using the snap-frozen PBMC. Briefly, DNA was isolated according to manufacturer’s instructions (Qiagen Isolation kit, Qiagen, Venlo, the Netherlands). Subsequently, TREC content was determined using quantitative PCR. For this purpose, a combination of two primers and a hydrolysis probe specific for the so-called δREC(TCRD)-ψJα(TCRA) TREC (sjTREC) were employed. TaqMan quantitative PCR was performed on 50 ng DNA in a 25 μl reaction mixture containing 700 nmol/l of each primer 5’-TCGTGAGAACGGTGAATGAAG-3’ and 5’-CCATGCTGACACCTCTGGTT-3’, 150 nmol/l of hydrolysis probe 5’-(FAM) CACGGTGATGCATAGGCACCTGC-3’ (TAMRA), and 12.5 μl 2× TaqMan Universal PCR Master Mix (Applied Biosystems, Nieuwerkerk a/d IJssel, The Netherlands). Quantification of the DNA amount in each sample was performed using a quantitative PCR of the single-copy albumin gene. All reactions were performed in duplicate, unless a threshold cycle (Ct) difference between replicates of >1.5 necessitated to repeat the PCR experiment. ΔCt was calculated by using the formula: Ct value TREC PCR – Ct value albumin PCR [13].

### Telomere length assay

Flow fluorescent *in situ* hybridization was performed to determine the relative telomere length of CD4^+^ and CD8^+^ T cells. For this purpose, the frozen PBMC were thawed and stained with either CD4-biotin (Beckman-Coulter, BV, Woerden, The Netherlands) or CD8-biotin (Biolegend, Europe BV, Uithoorn, the Netherlands) followed by staining with streptavidin-Cy5 (Biolegend). The PBMC were fixed and permeabilized (Invitrogen Life Technologies, Bleiswijk, The Netherlands) before the relative telomere length (RTL) was determined using the telomere PNA-kit/FITC (Zebra Bioscience BV, Enschede, The Netherlands). The sub cell line 1301 of CCRF-CEM, which is known to have long telomeres, was used to calculate the relative telomere length (RTL) of the CD4^+^ and CD8^+^ T cells [1,29].

### Statistical analysis

Patients not on RRT were compared to healthy controls on one hand and to patients receiving hemodialysis or peritoneal dialysis on the other hand using the Mann–Whitney test. For the TREC content and the RTL, a linear regression model was used to compare patients not on RRT to healthy controls. In addition, Spearman Rho correlation coefficients (Rs) were calculated to determine the strength of the association between the different ageing parameters and age for HC as well as ESRD patients not on RRT. All statistical tests were performed two-sided and a p-value of <0.05, was considered significant.

## Competing interests

All the authors declared no competing interests. This study was funded by the Dutch Kidney Foundation (KSPB.10.12).

## Authors’ contributions

RM: performed the experiments, statistical analysis and drafted the manuscript. NL: designed the study and drafted the manuscript. EdW: performed the experiments. AL: contributed in writing the manuscript. AvdS: performed some of the experiments. CB: contributed in writing the manuscript. WW: contributed in writing the manuscript and provided patient data. MB: designed the study and drafted the manuscript. All authors read and approved the final manuscript.
